# Inhalation of electronic cigarettes slightly affects lung function and inflammation in mice

**DOI:** 10.3389/ftox.2023.1232040

**Published:** 2023-09-04

**Authors:** Yuxing Dai, Kun Duan, Guangye Huang, Xuemin Yang, Xingtao Jiang, Jianwen Chen, Peiqing Liu

**Affiliations:** ^1^ Department of Pharmacology and Toxicology, School of Pharmaceutical Sciences, Sun Yat-Sen University, Guangzhou, Guangdong, China; ^2^ RELX Science Center, Shenzhen RELX Tech Co., Ltd., Shenzhen, China; ^3^ National and Local Joint Engineering Laboratory of Druggability and New Drugs Evaluation, Guangdong Engineering Laboratory of Druggability and New Drug Evaluation, School of Pharmaceutical Sciences, Sun Yat-sen University, Guangzhou, China

**Keywords:** electronic cigarette, lung function, cigarette, inflammation, toxicology

## Abstract

Electronic cigarettes have become increasingly popular, but the results of previous studies on electronic cigarette exposure in animals have been equivocal. This study aimed to evaluate the effects of electronic cigarette smoke (ECS) and cigarette smoke (CS) on lung function and pulmonary inflammation in mice to investigate whether electronic cigarettes are safer when compared to cigarettes. 32 specific pathogen-free BALB/c male mice were randomly grouped and exposed to fresh air (control), mint-flavored ECS (ECS1, 6 mg/kg), cheese-flavored ECS (ECS2, 6 mg/kg), and CS (6 mg/kg). After 3 weeks exposure to ECS or CS, we measured lung function (PIF and Penh) and blood oxygen saturation. The levels of TNF-α and IL-6 in the bronchoalveolar lavage fluid (BALF) and serum were measured using ELISA. HE staining was performed to observe the pathological changes in the lung tissues. The levels of IL-6 in BALF and serum, and TNF-α in BALF, were elevated similarly in the ECS and CS groups compared to the control group. Significant elevation was observed in serum TNF-α levels in the CS group. The total count of cells in BALF were increased after ECS1 exposure and CS exposure. PIF and oxygen saturation decreased, and Penh increased markedly in the CS group but not in the ECS groups. Compared with the ECS groups, mice in the CS group had widened lung tissue septa and increased inflammatory cell infiltration. However, we did not detect significant differences between mint-flavored and cheese-flavored e-cigarettes in our study. Overall, our findings suggested that both ECS and CS impair lung function and histopathology while promoting inflammation. In contrast, ECS has a less negative impact than CS.

## Introduction

Smoking is a major cause of morbidity and mortality, responsible for nearly 200 million deaths per year (Beaglehole et al., 2019). Tobacco smoking is the leading risk factor for serious lung diseases, including lung cancer and chronic obstructive pulmonary disease (Traboulsi et al., 2020). While traditional cigarette smoking has declined in recent years, the use of electronic cigarettes or e-cigarettes has increased dramatically (Bilano et al., 2015). E-cigarettes, also known as a type of electronic nicotine delivery system (ENDS), comprise a rechargeable battery, an atomizer, and liquid containing nicotine, solvent, and flavors, but do not contain natural tobacco. Electronic cigarette aerosols, produced by the heating and atomization of nicotine liquid via an atomizer, directly enter the lungs through the respiratory tract. The toxic and carcinogenic substances contained in cigarette smoke, including carbonyl compounds, volatile organic compounds, nitrosamines, and heavy metals, can still be detected in electronic cigarette aerosols, but their levels are much lower than those in cigarette smoke under the same average puffing conditions (Goniewicz et al., 2014).

E-cigarettes are marketed as a safer alternative to cigarettes, yet their health effects remain largely controversial (Rom et al., 2015). Reliable and direct support data on the safety of using electronic cigarettes for human health remains scanty (Wagner et al., 2017), and the effects of electronic cigarettes on human health have not been adequately clarified (Dinakar and O'Connor, 2016; Pisinger and Dossing, 2014). Due to a lack of preclinical scientific data in the field, it is critical to evaluate the *in vivo* safety of electronic cigarettes.

Previous research on e-cigarettes has focused on acute exposure, with studies showing that e-cigarette aerosol can induce inflammation, impair lung function and increase oxidative stress. Acute exposure to electronic cigarette vapor increases blood pressure (Fogt et al., 2016) and airway resistance (Vardavas et al., 2012). E-cigarette aerosols increase levels of monocyte chemoattractant protein-1 (MCP-1) and IL-6 in BALF after acute exposure to e-cigarettes (Lerner et al., 2015). Another study found that mice exposed to e-cigarettes for 6 h per day had higher IL-6 and TNF-α transcripts levels after 3 days. However, some studies have shown that e-cigarette exposure did not induce oxidative stress and cell death, and only a limited focus of inflammatory cell infiltration was observed on lung histology (Husari et al., 2016). It has been reported that the inflammation was not increased in mice exposed to e-cigarettes, but the lung function was decreased (Larcombe et al., 2017a).

More and more studies have examined the pulmonary effects of e-cigarettes short-term and long-term exposure. A *in vivo* study reported that 4 weeks exposure to e-cig vapor can induce inflammatory responses and adversely affect respiratory system mechanics (Glynos et al., 2018). Another study has shown that mice exposed to e-cigarette aerosol for 8 weeks did not have increased inflammation but did display decrements in parenchymal lung function while less worsening than combustive cigarette smoke (Larcombe et al., 2017b). It has also reported that exposure to inhaled nicotine-containing e-cigarette fluids for 4 months triggered adverse effects including cytokine expression, airway hyper-reactivity and lung tissue destruction (Garcia-Arcos et al., 2016).

E-cigarettes flavors have been founded to increase e-cigarettes use frequency among adolescent (Morean et al., 2018). However, it is widely validated that the flavorings used in e-cigarettes may exacerbate the adverse pulmonary effects induced by e-cigarettes. Thermal decomposition of flavoring compounds dominates formation of aldehydes during vaping, which toxic to human health (Khlystov and Samburova, 2016). Glynos et al. also have examined that the added flavor in e-cigs exacerbated the detrimental effects of e-cig vapor, inducing more severe health concerns.

Understanding the pulmonary impacts of e-cigarette use is critical to informing product regulations and user safety. This study aimed to determine the effects of short-term exposure to e-cigarette aerosol *versus* cigarette smoke on inflammation and lung function in mice. Before the products exposure, we collected the e-cigarettes aerosol and cigarette smoke for UPLC measurement to determine the equivalent nicotine concentration as exposure dose. The nicotine concentration, which served as the exposure dosage, was determined by UPLC analysis of the collected aerosol and smoke samples. BALB/c mice, aged 8 weeks, were selected to be exposed to either conventional cigarette smoke or e-cigarette aerosols with mint or cheese flavorings for 3 weeks. The pulmonary physiology, the levels of inflammatory cytokines in BALF and serum, and histopathological changes in the lung tissues were assessed. Our hypothesis postulates that e-cigarette inhalation results in heightened pulmonary inflammation, compromised lung function, and these adverse effects exhibit variations with diverse flavors.

## Materials and methods

### Animals

BALB/c mice were susceptible to cigarette smoke induced lung pathology and systemic inflammatory and fibrotic responses (H. Chen et al., 2021), so we purchased specific pathogen-free male BALB/c mice aged 8 weeks from Guangdong Medical Laboratory Animal Centre (China). They were kept in a 12-h light/dark cycle with a room temperature of 22°C and had *ad libitum* access to food and water. All animal procedures were approved by the Institutional Animal Care and Use Committee (IACUC), Sun Yat-Sen University (Approval No. SYSU-IACUC-2020-000259).

### Smoke collection and UPLC method for measuring nicotine

ESC or CS was generated by a smoking machine and following collected with a Cambridge filter in a whole-body chamber delivering the relevant smoke. The size of the chamber was 3.8 L, where the flow rate of the air pump was 2.0 L/min. Smoke delivery methods simulated the way humans inhale smoke. 55 mL of smoke was released in 3 s, paused for 27 s, and released again. Smoke was released at a rate of 2 times per minute. The total amount of smoke released was 110 mL/min and after 30 min the Cambridge filter was extracted with 10 mL of DMSO. The average nicotine content of ECS and CS was determined within 30 min using ultra-performance liquid chromatography (UPLC). An external standard approach was used for quantitative analysis. The mobile phase was made up of 10 mM ammonium acetate (A) and 0.3 mL/min acetonitrile (B). The analytical column was an ACQUITY UPLC HSS T3 2.1 × 100 mm × 1.8 µm. The injection volume and temperature of the column were 0.5 μL and 40°C, respectively. The following was the best gradient elution: from 0.0 to 6.0 min, A-B of 80:20; from 6.01 to 9.0 min, A-B of 10:90; from 9.01 to 12.0 min, A-B of 80:20. At a wavelength of 260 nm, UV chromatograms were detected.

### Design and dosage of exposure to ECS and CS design

The electronic cigarettes consumed by an adult contain approximately 40 mg of nicotine per day (Prochaska et al., 2022). For 60 kg, an average human body weight, the nicotine dosage for human is approximately 0.67 mg/kg (nicotine-by-weight). The Meeh-Rubner formula is a calculation formula of total body surface area most commonly used for small animals (TBSA = *k*W^2/3^, mean *k* value is 9.83) (Gouma et al., 2012). According to the Meeh-Rubner formula, the equivalent dosage for mice was approximately 6 mg/kg, thus the nicotine inhaled by mice weighing 0.02 kg is 12 mg. The results of the pre-experiment showed that the average concentration of nicotine was 0.1 mg/L. So, it can be calculated the equivalent nicotine volume inhaled by mice is approximately 1.2 L. The ventilation volume per minute of mice was 0.0217 L/min (Alexander et al., 2008). Thus, the mice exposed to the aerosol for 60 min could reach the nicotine dose of 6 mg/kg, which is equivalent to human nicotine consumption per day.

### The procedure for ECS and CS exposure

Thirty-two specific pathogen-free BALB/c mice were randomly divided into four groups. The control group was exposed to fresh air. The ECS1 group was exposed to mint-flavored electronic cigarette smoke (RELX Tech. Co., Ltd.). The ECS2 group was exposed to cheese-flavored electronic cigarette smoke (RELX Tech. Co., Ltd.). The CS group was exposed to cigarette smoke. Except for the control group, the other groups were exposed to smoke twice a day for 30 min each. The exposure lasted 3 weeks, 5 days a week. During smoke exposure, every four mice were exposed to one batch of smoke. Smoke is delivered in the same manner as the above collection method.

### Lung function tests

The lung function of mice after smoke exposure was assessed using a whole-body plethysmograph (EMKA Technologies, Paris, France) (Moriya et al., 2016). The mice were placed into chambers of the whole-body plethysmograph and recorded for 5 min to detect peak inspiratory flow (PIF) and decrease in enhanced pause (Penh) of the lungs.

### Blood oxygen saturation

Blood oxygen saturation in mice was monitored with a Mouse Ox Plus pulse oximeter (Chang et al., 2013) for mice, rats, and other small animals (Starr Life Sciences Corp., Oakmont, United States). The blood oxygen sensor was collared around the necks of mice, and the instrument was electrified to detect and record the blood oxygen saturation for 3 min after stabilization.

### BALF and serum acquire

Mice were anesthetized with 3% pentobarbital sodium (Van Hoecke et al., 2017). Further, the blood of the mice was acquired from the fundus venous plexus. The blood was centrifuged at 3,000 rpm for 10 min at 4°C to obtain serum. The mice were sacrificed, and their chests were opened fully to expose the lungs. 0.5 mL of 4°C normal saline was injected into the trachea and withdrawn slowly three times during 30 s. This procedure was repeated. The BALF obtained twice was mixed, and the total number of leukocytes in the BALF was counted using a hemocytometer under a microscope. After counting, the BALF was centrifuged and the cell-free supernatant was stored at −80°C until subsequent measurement of TNF-α and IL-6. The levels of IL-6 and TNF-α in the supernatant of BALF and serum were detected using ELISA kits.

### ELISA

Using mouse ELISA kits for IL-6 (9680008061219) and TNF-α (9680010061219) from ABclonal Biotechnology Co., Ltd. (Wuhan, China), the inflammatory cytokines IL-6 and TNF-α were quantified using enzyme-linked immunosorbent assay. The technical protocols are available on manufacturer’s website (https://abclonal.com.cn/mouse-elisa-kits/) To begin, place 100 ul of standard or test samples in each well and incubate for 2 h at 37°C before washing. Incubate for 1 h at 37°C with 100 ul Working Biotin Conjugate Antibody, then wash three times. Add 100 ul Working Streptavidin-HRR and incubate for 0.5 h at 37°C. Then wash 3 times. Incubate for 15–20 min at 37°C in the dark with 100 ul Substrate Solution. Add 50 ul Stop Solution to the mix. Detect the optical density within 5 min under 450 nm. Each test sample was loaded in triplicate, with the results being averaged to minimize random errors and ensure reliability.

### HE staining

The lung tissue, which not used to acquire for BALF, was fixed in formalin, embedded in paraffin, and sliced for hematoxylin-eosin staining with an HE staining kit (Boster Biological Technology Co., Ltd., Wuhan, China). Pathological changes in the lung tissues were observed under a microscope (Life Technologies Co., Ltd., New York, America), and each lung tissue sample was qualitatively analyzed.

### Statistical analysis

Means ± standard deviation was calculated and statistical analysis was performed using GraphPad Prism version 8.0 for Windows. Statistical differences of measurement data were ascertained by one-way analysis of variance, with Tukey’s multiple comparisons test. Count data were analyzed by Kruskal–Wallis test with Dunn’s multiple comparisons test. Statistical significance was set at *p*-value <0.05.

## Results

### Effects of ECS and CS on lung function

**FIGURE 1:**
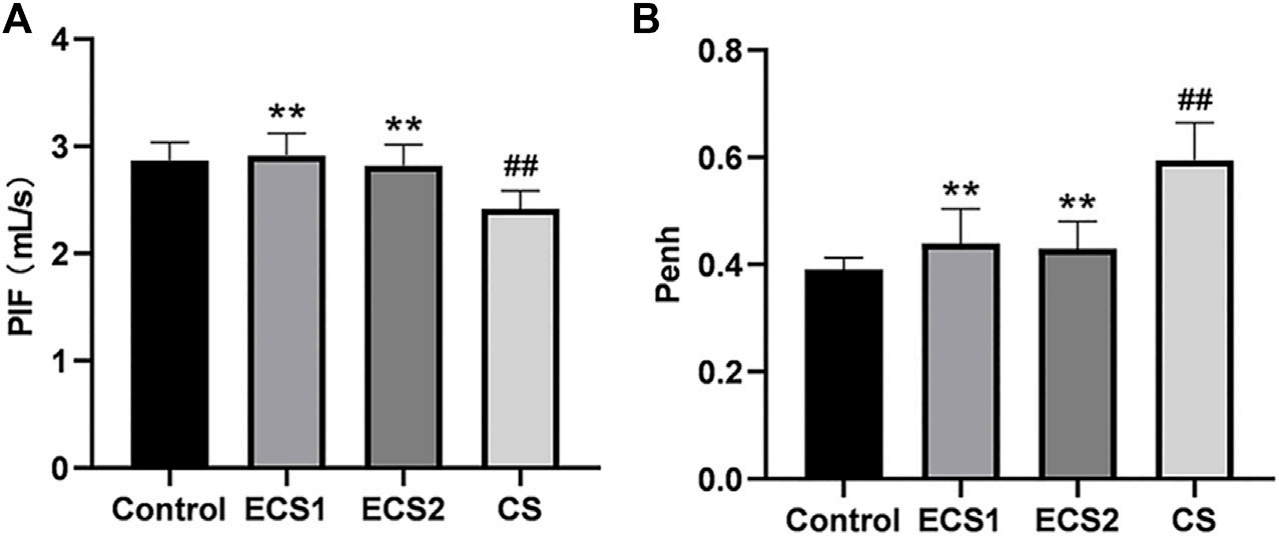
Effects of ECS and CS on PIF and Penh. PIF and Penh, as common indicators of lung function, were detected by a whole-body plethysmograph. Levels of PIF **(A)** and Penh **(B)** are presented (*n* = 8). Data are represented as mean ± SD. ** *p* < 0.01, significantly different to the CS group; ## *p* < 0.01, significantly different to the control group.

As the lung is exposed to smoke and is the main target organ for cigarettes, we assessed the lung function in the mice immediately after the completion of smoke exposure. The results of lung function testing showed PIF decreased and Penh increased significantly in the CS group compared to the control group (*p* < 0.01). Notably, in comparison to ECS group, PIF also fell drastically (*p* < 0.01), while Penh elevated significantly in CS-exposed mice (*p* < 0.01) (Figure 1).

### Effects of ECS and CS on blood oxygen saturation

**FIGURE 2:**
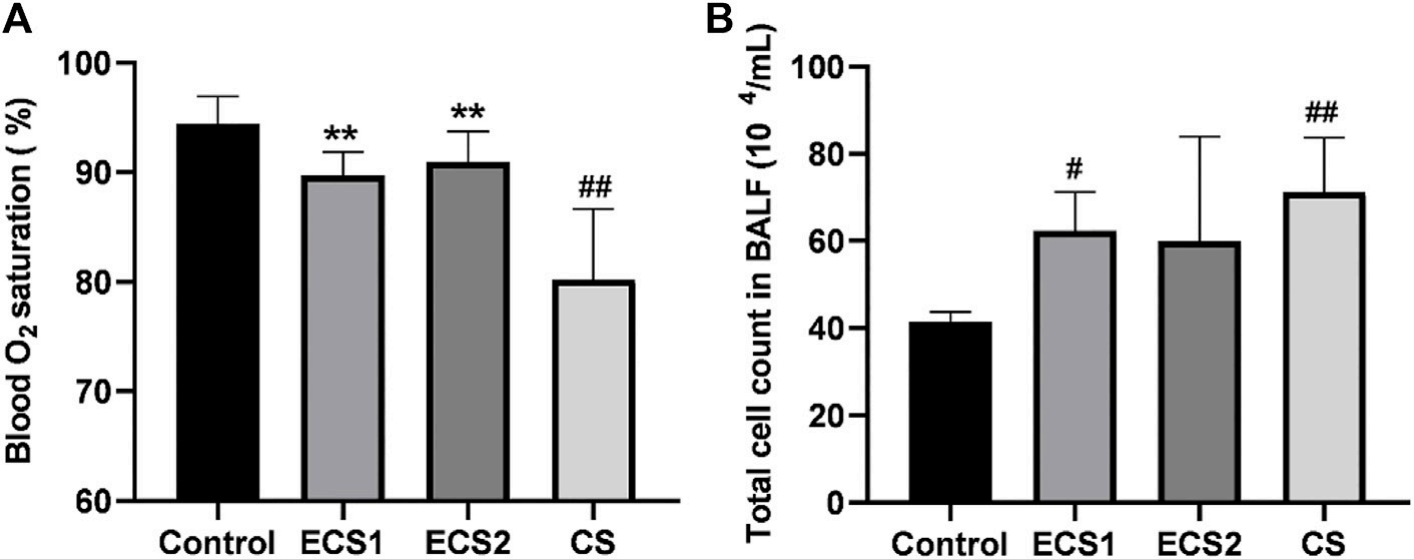
Effects of ECS and CS on blood oxygen saturation and the total cell count in BALF. Blood oxygen saturation was monitored with a Mouse Ox Plus pulse oximeter. The total cell count in BALF was counted under the microscope. Levels of blood oxygen saturation **(A)** and total count of cells in the BALF **(B)** are presented (*n* = 3–4). Data are represented as mean ± SD. ** *p* < 0.01, significantly different to the CS group; # *p* < 0.05, significantly different to the control group; ## *p* < 0.01, significantly different to the control group.

To further define lung function, the oxygen saturation of mice was examined. The study indicated that ESC exposure had no significant effect on blood oxygen saturation compared to air exposure, and the blood oxygen saturation in the CS group was markedly lower than that in the control group (*p* < 0.01). The blood oxygen saturation in each ECS group was significantly higher than that in the CS group (*p* < 0.01) (Figure 2).

### Effect of ECS and CS on the total count of cells in the BALF

Furthermore, we also measured the number of cells in the BALF. The results indicated that the total cell count in the BALF in the CS group was significantly higher than that in the control group (*p* < 0.01). The total count of cells in the ECS1 group was significantly higher compared to the control group (*p* < 0.05). Moreover, the total cell count in BALF in each ECS group decreased to a different degree than that in the CS group, but there was no significant difference (*p* > 0.05) (Figure 2).

### Effect of ECS and CS on the contents of TNF-α and IL-6 in BALF and serum

**FIGURE 3:**
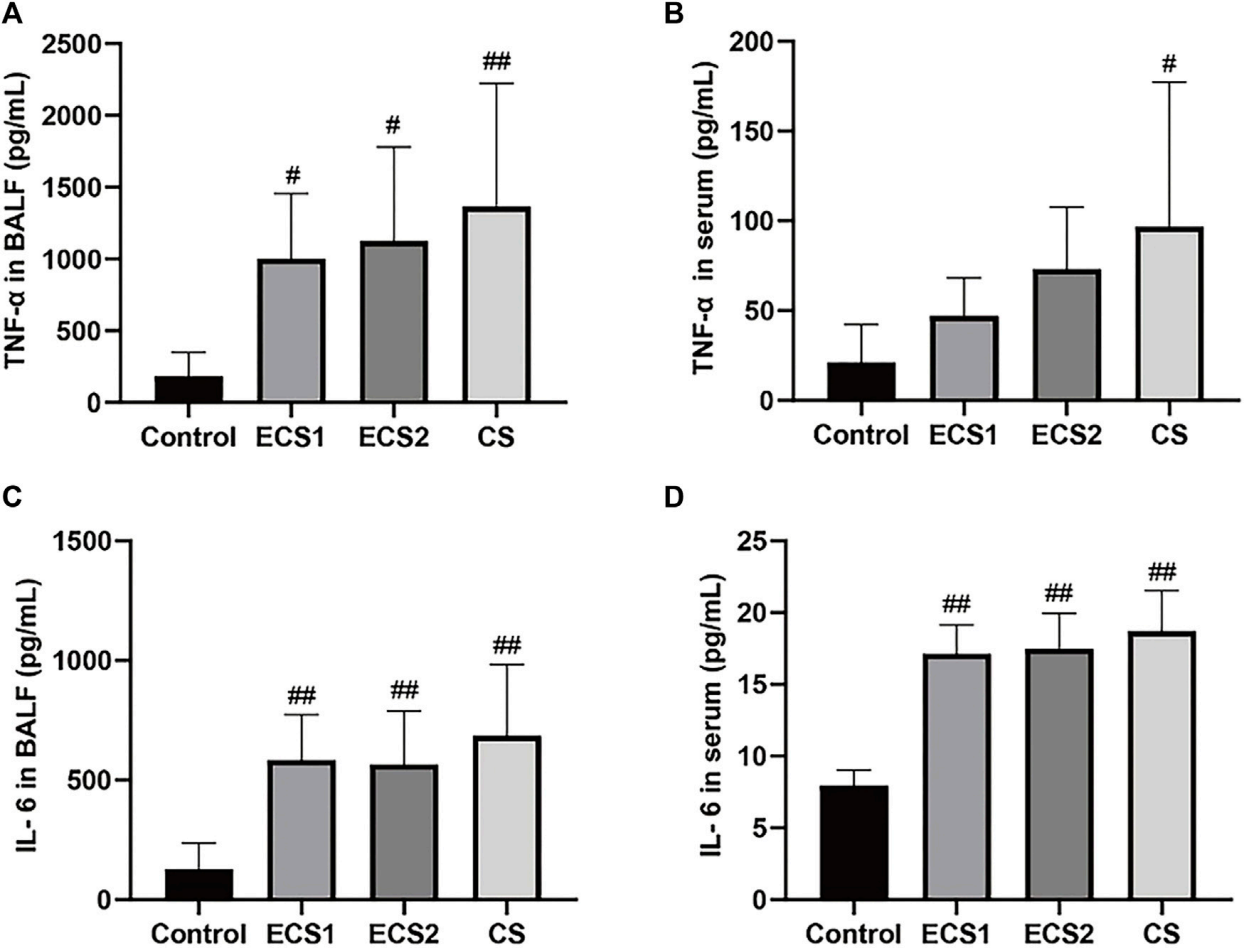
Effects of ECS and CS on TNF-α and IL-6 levels in BALF and serum. Levels of TNF-α in BALF**(A)**, TNF-α in serum **(B)**, IL-6 in BALF **(C)**, and IL-6 in serum **(D)** are presented (*n* = 3–4). Data are represented as mean ± SD. # *p* < 0.05, significantly different to the control group; ## *p* < 0.01, significantly different to the control group.

We examined the levels of inflammatory factors in serum and BALF to explore whether ECS caused an inflammatory response. When compared to the control group, the ECS groups had considerably greater levels of TNF-α in BALF (*p* < 0.05) and IL-6 in both BALF and serum (*p* < 0.01). TNF-α levels in BALF (*p* < 0.01) and serum (*p* < 0.05) were significantly higher in the CS group compared with the control group, as were IL-6 levels (*p* < 0.01). TNF-α and IL-6 values in BALF and serum were marginally lower in the ECS groups than in the CS group, but there was no significant difference (Figure 3).

### Effect of ECS and CS on HE staining in lung tissue

**FIGURE 4:**
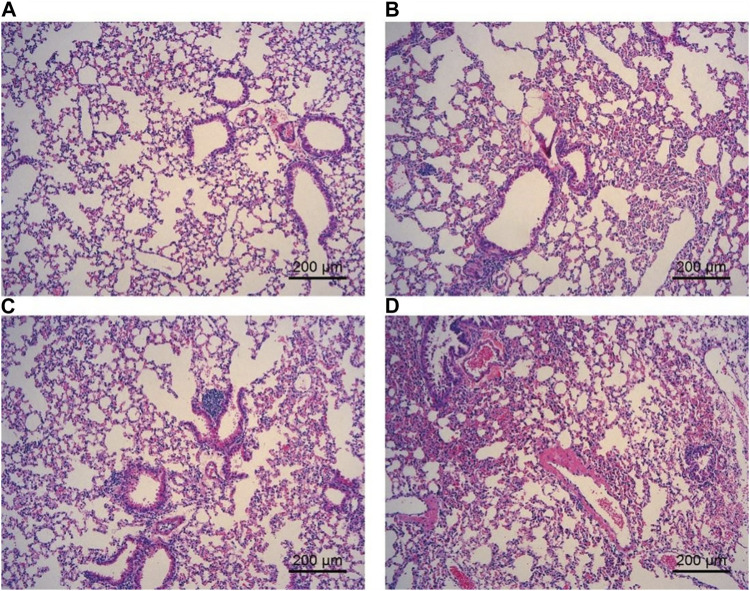
Effect of ECS and CS on HE staining of the lung tissue. The lung tissue was fixed, embedded in paraffin, and sliced for hematoxylin-eosin staining with an HE staining kit (*n* = 3–4). **(A)** represents the control group; **(B)** represents the ECS1 group; **(C)** represents the ECS2 group; **(D)** represents the CS group. Scale bar: 200 µm.

To observe the effect of ECS on the lung tissue structure, HE staining of lung tissue was performed. The analysis suggested that the lung tissue septum in animals exposed to cigarettes was significantly broadened, inflammatory cell infiltration was obvious, and increased local pulmonary hemorrhage and pulmonary interstitial congestion were found in comparison to animals exposed to air. Furthermore, the lung tissue septum was less widened, inflammatory cell infiltration was decreased, and pulmonary hemorrhage was less common in each ECS group than in the CS group (Figure 4).

## Discussion

The present study aimed to analyze the effects of short-term exposure to ECS and CS on lung function and inflammation in mice. Our findings suggested that ECS had a negative impact on pulmonary inflammation, whereas CS exerted more pronounced detrimental effect on lung function injur and inflammation. These results contribute to the ongoing controversy surrounding the potential benefits and harms of electronic cigarettes.

Electronic cigarettes have become increasingly popular recently, especially among teenagers. Consumption of electronic cigarettes may not be effective in promoting tobacco cessation (Kalkhoran and Glantz, 2016), but the reduction in tobacco smoking is indeed closely related to the use of electronic cigarettes in adolescent smokers with severe nicotine addiction (Selya et al., 2018). It is unclear whether they are beneficial or harmful for smokers and adolescents (Lam et al., 2014), The increasing prevalence of electronic cigarettes has raised serious concerns about their impact on public health (Pearson et al., 2012).

Lung function is an important indicator of lung health. As an overall indicator, the lung function in animals can directly represent the effect of ECS exposure. While many respiratory parameters were detected using EMKA system, most indicators lacked statistical differences to be included in our study. Penh is an important parameter that represents the gas exchange capacity and the severity of airway resistance (Ghorani et al., 2017). Penh reflects changes in lung function and airway resistance in conscious animals by using a whole-body thoracoscopic system (Halloy et al., 2004). Measurement of PIF is useful for interpreting inspiratory resistance to assess lung function in mice (Bentur et al., 2004). Decreased gas exchange capacity and increased airway resistance are typical clinical manifestations of decreased lung function (Jobse et al., 2013), and they are important diagnostic indicators of decreased lung function. Smoking is known to cause lung dysfunction (Zalokar, 1979). Regarding whether e-cigarettes impair lung function, several informative studies have addressed the effects of electronic cigarettes on lung health *in vivo* (Sussan et al., 2015; Husari et al., 2016), but they lack investigation into lung function mechanics. To address this problem, we evaluated the effect of ECS exposure on lung function. In this study, Penh and PIF values in mice were detected consciously. The results showed that CS exposure caused significant changes in PIF and Penh, but not in ECS exposure. These results suggest that exposure to CS is harmful and that it significantly affects lung function in mice. In contrast, exposure to ECS had slightly but no significant adverse effects on lung function in mice, but the damage to the lung function was significantly less than that of CS. Notably, more common lung function parameters should be evaluated again to expand the scope of endpoints including respiratory rate (RR), tidal volume (TV), peak expiratory flow (PEF), total lung volume (TLV), and forced expiratory volume (FEV).

Blood oxygen saturation is an important characteristic of the functioning of the respiratory and circulatory systems. The lungs are organs for oxygen exchange in mice. When lung function is impaired, the blood oxygen saturation may also change. The saturation changes with the severity of lung dysfunction in mice (Gross et al., 2020). In this study, we measured the blood oxygen saturation in conscious mice. The results showed that both CS and ECS caused a reduction in blood oxygen saturation, while ECS exposure exhibiting a significantly distinct effect on blood oxygen saturation compared to CS exposure. Taken together, lung dysfunction caused by exposure to CS but not ECS affects blood oxygen saturation in mice.

Lung injury in mice leads to an inflammatory response. Studies have shown that smoking reduces levels of the antioxidant glutathione (GSH) in the lung, which further activates redox-sensitive transcription factors including nuclear factor-κB (NF-κB) and activator protein-1 (AP-1), initiating a pulmonary inflammatory response (Rahman, 2003). During the inflammatory response, some cytokines influence the activation of inflammatory cells and they are recruited into the airways, which further leads to lung dysfunction (Szafran et al., 2020). It has been reported that passive smoking leads to slow weight gain, lung pathology in mice, and markedly elevated levels of serum IL-6 and TNF-α (Onishi et al., 2018; Romo et al., 2019). TNF-α has been identified as a key cytokine in the immunological response to CS exposure, regulating inflammation and promoting neutrophil recruitment via activating endothelial cells (Churg et al., 2003). In this research, we measured the total cell count in BALF, TNF-α, and IL-6 in the BALF and serum to study the effect of ECS exposure on lung inflammation. The results suggested that ECS exposure resulted in an increase in total cell count as well as increased inflammatory levels, but the effects were minor when compared to CS exposure. Therefore, exposure to both ECS and CS can cause inflammation in the body and lungs of mice, but the inflammation caused by CS is more serious. However, a more comprehensive evaluation of systemic inflammation including more inflammatory markers would provide a more detailed understanding. In addition to smoking, inhalation of other foreign substances can also cause lung injury and inflammation (Wang et al., 2019). Moreover, the severity of lung injury and inflammation is closely related to the amount of inhaled substances (Deb et al., 2012).

HE staining is an important method to determine pathological changes in tissue structure, and it is one of the most widely used methods for pathological biopsy (Zheng et al., 2019). The lung tissue of mice was sectioned and stained with an HE staining kit in this experiment. The results showed that exposure to both ECS and CS caused pathological changes in the lung tissue of mice, but the lung tissue lesions caused by exposure to CS were more serious than those caused by ECS. Therefore, these results suggest that both ECS and CS can cause changes in the lung tissue of mice, although CS is more toxic than ECS.

However, we recognize that our evaluation of total BALF cellularity and images of HE-stained histological lung sections provides a summary-level survey, which may limit our ability to make specific connections between BALF cytokine levels and the mechanisms of inflammation on pulmonary function. A more comprehensive analysis of BALF cellular constituents and immunohistochemical evaluation of lung sections would provide a clearer understanding of the potential mechanistic linkages to changes in pulmonary function.

One limitation of our study is that it did not address the potential impact of different flavors on lung function and inflammation. Previous research has suggested that certain flavors may have differential effects on lung health. Bahl et al. (2012) first suggested that cytotoxicity of e-cigarette liquids (e-liquids) was related to flavors. However, Misra et al. (2014) indicated that e-liquids or collected aerosol produced any meaningful toxic effects *in vitro*. This controversy may be driven form cell lines. As in our mice model, we did not find any significant differences between different flavors in terms of lung function and inflammation. Further research is needed to explore the role of flavorings in electronic cigarette toxicity using more widely marketed flavorings.

Another important aspect to consider is the aerosol components of electronic and traditional cigarette smoke, which may contribute to the differential lung effects observed in our study. While both ECS and CS contain nicotine, the chemical composition of their aerosols differs significantly (Li et al., 2020). The aerosol from the e-cigarette is compositionally less complex than conventional cigarette smoke, containing significantly lower levels of toxicants (Margham et al., 2016). This difference in composition may partially explain the observed differences in lung function and inflammation between ECS and CS exposure in our study.

In this study, we used a whole-body exposure chamber as opposed to nose-only exposure system, which may introduce some uncertainty due to the grooming and cleaning behaviors of rodents between exposures. This behavior could result in incidental ingestion of cigarette smoke and related substances, in addition to inhalation exposures. Studies have reported that whole-body exposure systems may lead to a combination of oral and inhalation exposures in rodents (L.-C. Chen and Lippmann, 2015), which could potentially confound the interpretation of our findings. Despite this limitation, however, whole-body exposure systems have been widely used in inhalation toxicology studies since it could mimic real-world exposure behavior, where humans are also exposed to both inhalation and incidental ingestion of environmental pollutants (Serré et al., 2021). Compared to whole-body exposure, the use of a nose-only exposure system could help to minimize confounding factors related to incidental ingestion, allowing for a more precise assessment of inhalation-specific effects on pulmonary function (Kogel et al., 2021).

Our study evaluated the toxicity of ECS and CS using equivalent dosing, which may not accurately represent real-world smoking patterns because smokers tailor their smoking to different products and toxicologic effects change with different smoking profiles (Marian et al., 2009). It is essential to consider that electronic cigarette users and traditional smokers may exhibit differences in smoking patterns, such as puff frequency, duration, and intensity. Moreover, the nicotine content and other toxic matters also vary in electronic cigarettes with different brands, model numbers, and flavors, inducing variability in potential human exposures (Stratton et al., 2018). These variations in smoking behavior could potentially alter the toxicity of the aerosols and their impact on lung function and inflammation. Future studies should investigate the effects of different smoking patterns on lung health to better understand the potential risks associated with electronic cigarette use in comparison to traditional smoking.

In terms of the global public health impact, our findings suggest that electronic cigarettes may be less harmful to lung health than conventional cigarettes, at least in the context of short-term exposure. This information could be useful in guiding public health policies and strategies aimed at reducing the harm associated with tobacco smoking. However, it is crucial to emphasize that our study was conducted in mice, and the results may not directly translate to humans. Additional research is needed to confirm the effects of electronic cigarette use on lung function and inflammation in human populations, as well as to identify any potential long-term consequences.

Overall, our study provides new insights into the effects of short-term exposure to ECS and CS on lung function and inflammation in mice. While both ECS and CS exposure can induce lung dysfunction and inflammation, the severity of these effects is considerably higher following CS exposure. Our findings contribute to the ongoing debate surrounding the potential benefits and harms of electronic cigarettes, highlighting the need for further research to better understand their long-term consequences and inform public health policies.

## Conclusion

The study compared the effects of short-term exposure to ECS with mint and cheese flavors and CS in mice. Our findings revealed that exposure to both electronic cigarettes and conventional cigarettes negatively impacts lung physiology. However, the lung function and blood oxygen saturation in the group of electronic cigarettes improved compared to those in the conventional cigarette group. There is no significant difference between mint-flavored and cheese-flavored electronic cigarettes in our model. Notably, our findings seem supported that the harmful effects of electronic cigarettes were less than those of conventional cigarettes in mice.

## Data Availability

The original contributions presented in the study are included in the article/Supplementary Material, further inquiries can be directed to the corresponding authors.
